# Supplementary material for: Daily and Seasonal Activity Patterns of Plateau Pikas (Ochotona curzoniae) on the Qinghai–Tibet Plateau, China, and Their Relationship with Weather Condition
Source: Animals (Basel). 2023 May 18;13(10):1689. doi: 10.3390/ani13101689 (PMC10215905; doi:10.3390/ani13101689)
Supplement: Supplementary file 1 [file animals-13-01689-s001.zip › animals-2272871-supplementary.pdf]

**Table S1. Correlation Matrices.** Matrices displaying correlations among all predictor variables in cold season (n = 3456). Models based on combinations of highly correlated predictors (Spearman's r > 0.5) were not considered. (WS- wind speed; TEM - air temperature; RH - relative air humidity; PRE - precipitation; DL - day length)

| | **WS** | **TEM** | **RH** | **PRE** | **DL** |
|---|---|---|---|---|---|
| **WS** | 1 | | | | |
| **TEM** | 0.409 | 1 | | | |
| **RH** | -0.337 | 0.075 | 1 | | |
| **PRE** | 0.218 | 0.126 | 0.264 | 1 | |
| **DL** | 0.153 | 0.644 | 0.436 | 0.303 | 1 |

**Table S2. Correlation Matrices.** Matrices displaying correlations among all predictor variables in warm season (n = 2592). Models based on combinations of highly correlated predictors (Spearman's r > 0.5) were not considered. (WS- wind speed; TEM - air temperature; RH - relative air humidity; PRE - precipitation; DL - day length)

| | **WS** | **TEM** | **RH** | **PRE** | **DL** |
|---|---|---|---|---|---|
| **WS** | 1 | | | | |
| **TEM** | 0.663 | 1 | | | |
| **RH** | -0.388 | -0.631 | 1 | | |
| **PRE** | -0.167 | -0.147 | 0.363 | 1 | |
| **DL** | 0.185 | 0.451 | 0.108 | 0.128 | 1 |

**Table S3. Candidate models.** Predictors in each candidate model include wind speed (WS), air temperature (TEM), relative air humidity (RH), precipitation (PRE), daylength (DL).

| **Analysis 1: in the cold season** | |
|---|---|
| **Model** | **Predictors** |
| 1 | WS |
| 2 | WS, TEM |
| 3 | WS, RH |
| 4 | WS, PRE |
| 5 | WS, DL |
| 6 | WS, TEM, RH |
| 7 | WS, TEM, PRE |
| 8 | WS, RH, PRE |
| 9 | WS, RH, DL |
| 10 | WS, PRE, DL |
| 11 | WS, TEM, RH, PRE |
| 12 | TEM |
| 13 | TEM, RH |
| 14 | TEM, PRE |
| 15 | TEM, RH, PRE |
| 16 | RH |
| 17 | RH, PRE |
| 18 | RH, DL |
| 19 | RH, PRE, DL |
| 20 | PRE |
| 21 | PRE, DL |
| 22 | DL |
| 23 | NULL |
| **Analysis 2: in the warm season** | |
| **Model** | **Predictors** |

| | |
|---|---|
| 1 | WS |
| 2 | WS, RH |
| 3 | WS, PRE |
| 4 | WS, DL |
| 5 | WS, RH, PRE |
| 6 | WS, RH, DL |
| 7 | WS, PRE, DL |
| 8 | WS, RH, PRE, DL |
| 9 | TEM |
| 10 | TEM, PRE |
| 11 | TEM, DL |
| 12 | TEM, PRE, DL |
| 13 | RH |
| 15 | RH, PRE |
| 16 | RH, DL |
| 17 | RH, PRE, DL |
| 18 | PRE |
| 19 | PRE, DL |
| 20 | DL |
| 21 | NULL |

**Table S4. Coefficients and 95% confidence intervals (CIs) of activity overlap among different months. *p*-values (*p*) obtained by testing whether the two sets of observations come from the same circular distribution are also reported. Statistically significant values of effect sizes and probabilities ($p < 0.05$) are in bold type.**

| Comparison | Difference | SE | W | *p* |
|---|---|---|---|---|
| January vs. February | 0.016 | 0.019 | 0.697 | 0.4038 |
| January vs. March | -0.223 | 0.019 | 132.002 | **0.0000** |
| January vs. April | -0.118 | 0.026 | 20.495 | **0.0000** |
| January vs. May | -0.128 | 0.040 | 10.296 | **0.0013** |
| January vs. June | -0.182 | 0.024 | 55.771 | **0.0000** |
| January vs. July | -0.146 | 0.026 | 32.286 | **0.0000** |
| January vs. August | -0.261 | 0.021 | 153.263 | **0.0000** |
| January vs. September | -0.199 | 0.030 | 44.210 | **0.0000** |
| January vs. October | -0.060 | 0.019 | 10.178 | **0.0014** |
| January vs. November | -0.065 | 0.022 | 8.512 | **0.0035** |
| January vs. December | -0.034 | 0.019 | 3.132 | 0.0768 |
| February vs. March | -0.239 | 0.020 | 144.963 | **0.0000** |
| February vs. April | -0.133 | 0.026 | 25.723 | **0.0000** |
| February vs. May | -0.144 | 0.040 | 12.845 | **0.0003** |
| February vs. June | -0.198 | 0.025 | 64.069 | **0.0000** |
| February vs. July | -0.162 | 0.026 | 38.653 | **0.0000** |
| February vs. August | -0.277 | 0.022 | 166.152 | **0.0000** |
| February vs. September | -0.215 | 0.030 | 50.551 | **0.0000** |
| February vs. October | -0.076 | 0.019 | 15.478 | **0.0001** |
| February vs. November | -0.081 | 0.023 | 12.704 | **0.0004** |

| | | | | |
|---|---|---|---|---|
| February vs. December | -0.050 | 0.020 | 6.377 | **0.0116** |
| March vs. April | 0.106 | 0.027 | 15.620 | **0.0001** |
| March vs. May | 0.095 | 0.040 | 5.486 | **0.0192** |
| March vs. June | 0.041 | 0.025 | 2.699 | 0.1004 |
| March vs. July | 0.077 | 0.026 | 8.444 | **0.0037** |
| March vs. August | -0.038 | 0.022 | 3.015 | 0.0825 |
| March vs. September | 0.024 | 0.031 | 0.616 | 0.4324 |
| March vs. October | 0.163 | 0.020 | 67.190 | **0.0000** |
| March vs. November | 0.158 | 0.023 | 46.497 | **0.0000** |
| March vs. December | 0.189 | 0.020 | 85.626 | **0.0000** |
| April vs. May | -0.011 | 0.044 | 0.059 | 0.8073 |
| April vs. June | -0.064 | 0.030 | 4.445 | **0.0350** |
| April vs. July | -0.029 | 0.032 | 0.821 | 0.3649 |
| April vs. August | -0.144 | 0.028 | 26.433 | **0.0000** |
| April vs. September | -0.082 | 0.035 | 5.391 | **0.0202** |
| April vs. October | 0.057 | 0.026 | 4.748 | **0.0293** |
| April vs. November | 0.052 | 0.029 | 3.297 | 0.0694 |
| April vs. December | 0.083 | 0.027 | 9.699 | **0.0018** |
| May vs. June | -0.054 | 0.043 | 1.545 | 0.2138 |
| May vs. July | -0.018 | 0.044 | 0.167 | 0.6832 |
| May vs. August | -0.133 | 0.041 | 10.363 | **0.0013** |
| May vs. September | -0.071 | 0.046 | 2.322 | 0.1275 |
| May vs. October | 0.068 | 0.040 | 2.865 | 0.0905 |
| May vs. November | 0.063 | 0.042 | 2.269 | 0.1320 |

| | | | | |
|---|---|---|---|---|
| May vs. December | 0.094 | 0.041 | 5.384 | **0.0203** |
| June vs. July | 0.036 | 0.030 | 1.386 | 0.2392 |
| June vs. August | -0.079 | 0.026 | 9.026 | **0.0027** |
| June vs. September | -0.017 | 0.034 | 0.259 | 0.6108 |
| June vs. October | 0.122 | 0.025 | 24.213 | **0.0000** |
| June vs. November | 0.117 | 0.027 | 18.098 | **0.0000** |
| June vs. December | 0.148 | 0.025 | 34.413 | **0.0000** |
| July vs. August | -0.115 | 0.028 | 17.228 | **0.0000** |
| July vs. September | -0.053 | 0.035 | 2.293 | 0.1299 |
| July vs. October | 0.086 | 0.026 | 10.869 | **0.0010** |
| July vs. November | 0.081 | 0.029 | 7.998 | **0.0047** |
| July vs. December | 0.112 | 0.026 | 17.835 | **0.0000** |
| August vs. September | 0.062 | 0.032 | 3.854 | **0.0496** |
| August vs. October | 0.201 | 0.022 | 87.272 | **0.0000** |
| August vs. November | 0.196 | 0.025 | 63.588 | **0.0000** |
| August vs. December | 0.227 | 0.022 | 106.305 | **0.0000** |
| September vs. October | 0.139 | 0.030 | 21.085 | **0.0000** |
| September vs. November | 0.134 | 0.033 | 16.989 | **0.0000** |
| September vs. December | 0.165 | 0.031 | 28.997 | **0.0000** |
| October vs. November | -0.005 | 0.023 | 0.046 | 0.8296 |
| October vs. December | 0.026 | 0.020 | 1.690 | 0.1936 |
| November vs. December | 0.031 | 0.023 | 1.759 | 0.1848 |

**Table S5. Estimates of percentage of time active (activity level) for six time periods of cold and warm season from the distribution of camera trapping photos over the daily cycle.**

| Season | Time periods | n | Activity level | | |
|---|---|---|---|---|---|
| | | | Estimate | SE | 95%CI |
| cold | Nighttime | 24 | 0.023 | 0.004 | 0.012-0.026 |
| | Before sunrise | 28 | 0.053 | 0.007 | 0.041-0.062 |
| | After sunrise | 158 | 0.055 | 0.003 | 0.050-0.059 |
| | Daytime | 2821 | 0.268 | 0.005 | 0.262-0.277 |
| | Before sunset | 142 | 0.057 | 0.004 | 0.054-0.064 |
| | After sunset | 115 | 0.064 | 0.006 | 0.056-0.072 |
| | total | 3288 | 0.269 | 0.006 | 0.255-0.272 |
| warm | Nighttime | 40 | 0.015 | 0.029 | 0.009-0.017 |
| | Before sunrise | 49 | 0.035 | 0.003 | 0.030-0.038 |
| | After sunrise | 134 | 0.049 | 0.003 | 0.044-0.053 |
| | Daytime | 3611 | 0.337 | 0.007 | 0.328-0.347 |
| | Before sunset | 335 | 0.060 | 0.004 | 0.055-0.065 |
| | After sunset | 210 | 0.066 | 0.004 | 0.059-0.071 |
| | total | 4379 | 0.419 | 0.011 | 0.401-0.429 |

**Table S6. Coefficients and 95% confidence intervals (CIs) of activity overlap among six time periods. *p*-values (*p*) obtained by testing whether the two sets of observations come from the same circular distribution are also reported. Statistically significant values of effect sizes and probabilities ($p < 0.05$) are in bold type.**

| Season | Comparison | Difference | SE | W | *p* |
|---|---|---|---|---|---|
| cold | Nighttime vs. Before sunrise | 0.177 | 0.044 | 16.298 | **0.0001** |
| | Nighttime vs. After sunrise | 0.175 | 0.043 | 16.267 | **0.0001** |
| | Nighttime vs. Daytime | -0.038 | 0.044 | 10.756 | **0.0001** |
| | Nighttime vs. Before sunset | 0.173 | 0.044 | 15.745 | **0.0001** |
| | Nighttime vs. After sunset | 0.166 | 0.044 | 14.424 | **0.0001** |
| | Before sunrise vs. After sunrise | -0.002 | 0.008 | 0.066 | 0.7977 |
| | Before sunrise vs. Daytime | -0.215 | 0.009 | 621.646 | **0.0000** |
| | Before sunrise vs. Before sunset | -0.005 | 0.008 | 0.359 | 0.5490 |
| | Before sunrise vs. After sunset | -0.011 | 0.009 | 1.561 | 0.2115 |
| | After sunrise vs. Daytime | -0.213 | 0.006 | 1261.508 | **0.0000** |
| | After sunrise vs. Before sunset | -0.003 | 0.005 | 0.335 | 0.5625 |
| | After sunrise vs. After sunset | -0.009 | 0.006 | 2.046 | 0.1526 |
| | Daytime vs. Before sunset | 0.211 | 0.006 | 1142.532 | **0.0000** |
| | Daytime vs. After sunset | 0.204 | 0.008 | 709.269 | **0.0000** |
| | Before sunset vs. After sunset | -0.007 | 0.007 | 0.960 | 0.3273 |
| warm | Nighttime vs. Before sunrise | 0.111 | 0.029 | 14.917 | **0.0001** |
| | Nighttime vs. After sunrise | 0.098 | 0.029 | 11.426 | **0.0007** |
| | Nighttime vs. Daytime | -0.190 | 0.030 | 41.127 | **0.0000** |
| | Nighttime vs. Before sunset | 0.087 | 0.029 | 8.941 | **0.0028** |
| | Nighttime vs. After sunset | 0.080 | 0.029 | 7.704 | **0.0055** |

| | | | | |
|---|---|---|---|---|
| Before sunrise vs. After sunrise | -0.014 | 0.004 | 10.131 | **0.0015** |
| Before sunrise vs. Daytime | -0.301 | 0.008 | 1430.396 | **0.0000** |
| Before sunrise vs. Before sunset | -0.025 | 0.005 | 25.222 | **0.0000** |
| Before sunrise vs. After sunset | -0.031 | 0.005 | 36.593 | **0.0000** |
| After sunrise vs. Daytime | -0.287 | 0.008 | 1233.992 | **0.0000** |
| After sunrise vs. Before sunset | -0.011 | 0.005 | 4.352 | 0.0370 |
| After sunrise vs. After sunset | -0.017 | 0.005 | 9.927 | **0.0016** |
| Daytime vs. Before sunset | 0.276 | 0.009 | 1053.250 | **0.0000** |
| Daytime vs. After sunset | 0.270 | 0.009 | 982.799 | **0.0000** |
| Before sunset vs. After sunset | -0.006 | 0.006 | 1.070 | 0.3008 |

**Table S7.** Relative support for models of activity level of plateau pika in the cold and warm season. Models are ranked in order of increasing AIC values (Akaike's information criterion). Log(L) denotes likelihood. df is the degree of freedom. ΔAIC is the difference between the indicated model and the best model (the model with lowest AIC). Unsupported models (ΔAIC > 4) are not shown. Akaike weight ($W_i$) and estimate of variation ($r^2$) are reported for each model. (TEM - air temperature; WS – wind speed; PRE – precipitation; RH - relative air humidity; DL – day length).

| Season | Model | df | Log(L) | AIC | △AIC | Wi | $r^2$ |
|---|---|---|---|---|---|---|---|
| Cold | PRE+WS***+RH+TEM*** | 19.12 | -950.42 | 1839.34 | 0 | 0.75 | 0.51 |
| | WS***+PRE+TEM*** | 17.23 | -953.57 | 1841.82 | 2.48 | 0.22 | 0.19 |
| Warm | PRE***+TEM***+DL | 9.11 | -1055.549 | 2029.16 | 0 | 0.98 | 0.75 |
| | TEM***+DL | 7.09 | -1061.437 | 2036.91 | 3.75 | 0.02 | 0.18 |

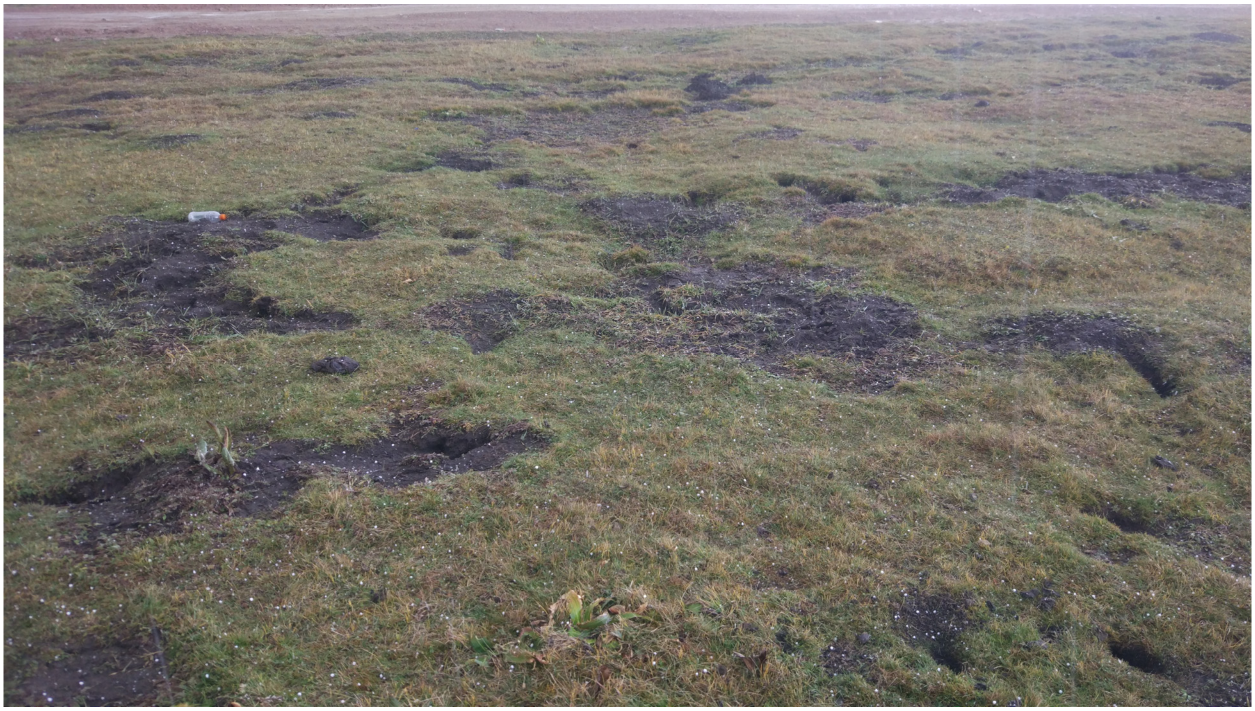

**Figure S1. Habitat of plateau pika, the photo was taken by Rui Zhou in Maqu County, Gansu Province, China.**
